# An integrative analysis of gene expression and molecular interaction data to identify dys-regulated sub-networks in inflammatory bowel disease

**DOI:** 10.1186/s12859-016-0886-z

**Published:** 2016-01-19

**Authors:** Daniele Muraro, Alison Simmons

**Affiliations:** Weatherall Institute of Molecular Medicine, University of Oxford, John Radcliffe Hospital, OX3 9DS Oxford, UK

**Keywords:** Inflammatory bowel disease, Molecular interaction network, Evolutionary algorithm

## Abstract

**Background:**

Inflammatory bowel disease (IBD) consists of two main disease-subtypes, Crohn’s disease (CD) and ulcerative colitis (UC); these subtypes share overlapping genetic and clinical features. Genome-wide microarray data enable unbiased documentation of alterations in gene expression that may be disease-specific. As genetic diseases are believed to be caused by genetic alterations affecting the function of signalling pathways, module-centric optimisation algorithms, whose aim is to identify sub-networks that are dys-regulated in disease, are emerging as promising approaches.

**Results:**

In order to account for the topological structure of molecular interaction networks, we developed an optimisation algorithm that integrates databases of known molecular interactions with gene expression data; such integration enables identification of differentially regulated network modules. We verified the performance of our algorithm by testing it on simulated networks; we then applied the same method to study experimental data derived from microarray analysis of CD and UC biopsies and human interactome databases. This analysis allowed the extraction of dys-regulated subnetworks under different experimental conditions (inflamed and uninflamed tissues in CD and UC). Optimisation was performed to highlight differentially expressed network modules that may be common or specific to the disease subtype.

**Conclusions:**

We show that the selected subnetworks include genes and pathways of known relevance for IBD; in particular, the solutions found highlight cross-talk among enriched pathways, mainly the JAK/STAT signalling pathway and the EGF receptor signalling pathway. In addition, integration of gene expression with molecular interaction data highlights nodes that, although not being differentially expressed, interact with differentially expressed nodes and are part of pathways that are relevant to IBD. The method proposed here may help identifying dys-regulated sub-networks that are common in different diseases and sub-networks whose dys-regulation is specific to a particular disease.

**Electronic supplementary material:**

The online version of this article (doi:10.1186/s12859-016-0886-z) contains supplementary material, which is available to authorized users.

## Background

Inflammatory bowel disease (IBD), including ulcerative colitis (UC) and Crohn’s disease (CD), arises from a breakdown in the normally symbiotic relationship between intestinal microflora and mucosa in individuals with a given genetic background. A recent Genome Wide Association Study has revealed 163 susceptibility loci that may contribute to development of IBD [1].

Genetic diseases are often believed to be caused by the combined alterations of genes that influence a common component of the cellular system [2]. Patterns in differential gene expression between healthy and diseased states may highlight pathological pathways; however, they are not informative about what upstream molecular interactions and signaling events control such gene expression changes [3–5]. Integration of gene expression data with databases of known molecular interactions may provide several advantages in terms of uncovering functional pathways driving disease specific expression signatures, identification of ‘hidden nodes’ that, although not being differentially expressed, may play an important role in connecting differentially expressed genes, and increased statistical robustness since differential expression is evaluated at a network level rather than for each gene individually [2, 3, 6].

Although the modularity of cellular systems is widely accepted, there is as yet no agreement on a unique mathematical definition of a network module. In the context of disease networks, network modules are typically defined as subsets of highly interconnected genes showing a significant overall differential expression in disease as compared with control cells [2].

If the network is modular, then a group of nodes that are more closely associated with themselves than with the rest of the network, called communities, should define network modules with similar biological roles [7]. Since the search for optimal sub-networks cannot exhaustively explore the search space, optimisation requires a heuristic strategy [5, 8]. One such approach may be using evolutionary algorithms which are well-suited for global optimisation strategies in discrete search spaces [9].

Evolutionary algorithms are optimisation algorithms based on the Darwinian principle of natural selection [10]. The quantities to be optimised are described as individuals that are sampled within a population. Each individual is associated with a fitness function which is optimised through natural selection (survival of the fittest).

In this article we propose an evolutionary algorithm whose aim is to identify overlapping and non-overlapping disease modules with highest differential expression under two conditions.

Several algorithms have previously been developed to optimise differentially regulated subnetworks from transcriptomic or phosphoproteomic data [5, 8, 11]. Other approaches have focused on identifying community structure in general complex networks [12–14]. However, these two methodologies define genetic representations and optimisation operators that do not integrate with one another. In fact, the operators used in algorithms for community detection allow the identification of network clusters, but do not enable selection of optimal subnetworks. Conversely, the algorithms proposed by Ideker et al. [5], Klammer et al. [8] and Chuang et al. [11] do not account for community structure and the genetic algorithm proposed by Klammer et al. does not account for maintenance of network connectivity. In our study we integrate differential expression and community detection by defining evolutionary optimisation operators generating connected subnetwork communities.

The algorithm performance was verified on simulated networks with topological features resembling the ones of experimental networks. Optimisation was then applied to real networks that were built by integrating molecular interaction databases with microarray data obtained from single endoscopy pinch biopsies from areas of uninflamed or inflamed mucosa in patients with CD and UC [4]. Subnetworks with statistically significant differential expression were identified by varying subnetwork size; in addition, functional analysis of the most frequently identified nodes showed crosstalk among enriched pathways and several hidden nodes. Several overlapping and non-overlapping differentially expressed subnetworks in CD and UC patients were detected, highlighting small overlap among the most frequently identified nodes between inflamed and uninflamed tissues. These optimal solutions included cross-talk among enriched pathways, mainly the JAK/STAT signalling pathway, EGF receptor signalling pathway, Gonadotropin releasing hormone receptor pathway and p38 MAPK pathway.

## Methods

### Optimisation algorithm

**Fig. 1:**
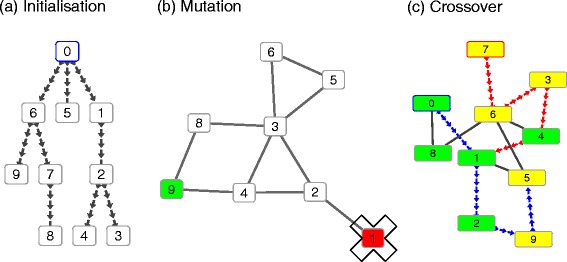
Schematic of our evolutionary algorithm. Individuals are defined as connected subnetworks and are initialised using a depth first search algorithm; the selection path is represented with directed arrows starting from an initial node highlighted with a blue border (**a**). Optimisation is performed by applying mutation and crossover operators. The mutation operator randomly changes a node by maintaining network connectivity (**b**). The crossover operator merges two parent subnetworks, represented with green and yellow nodes, into a connected one and generates two new subnetworks applying a depth first search algorithm to an initial, randomly selected node; the selection paths generating the offspring are represented with blue and red directed arrows starting from initial nodes highlighted respectively with blue and red borders (**c**)

In this section we outline the operators of our optimisation algorithm. These operators are defined for the purpose of identifying connected dysregulated protein interaction subnetworks integrating molecular interaction data with transcriptomic data generated from CD and UC patients. Since these diseases share partially overlapping genetic features, the focus of our algorithm is to highlight which active network modules are common to the two disease subtypes and which are specific to a particular disease subtype. The algorithm input consists of a network of known protein interactions and of the z-scores calculated from the *p*-values of two lists of differentially expressed genes; the latter are derived from biopsies of patients affected by CD against controls (healthy patients) and biopsies of patients affected by UC against controls (see section “Data pre-processing” for more details). The network is defined by associating a gene and its corresponding protein product with each node, whereas each edge represents an interaction between two proteins. Two z-scores are assigned to each node representing its differential expression under two conditions. Our evolutionary algorithm is based on an adaptation of the operators of a genetic algorithm on networks where the genetic operators have been modified in order to maintain connectivity of the optimised subnetworks (active network modules). Optimisation is performed by sampling a set of subnetworks, where each subnetwork is defined as an ‘individual’ and the set of subnetworks as a ‘population’, assigning a quality score to each subnetwork (‘fitness function’) and applying evolutionary algorithm operators (‘crossover’, ‘mutation’, ‘selection’) that we adapted to optimisation of networks as described below. A schematic diagram outlining these operators is presented in Fig. 1.

#### Individual representation and selection

Each individual of the population is defined as a subnetwork with a single connected component and predefined size. A tournament selection is performed as implemented by Deb et al. [15] including elitism on the best two individuals.

#### Fitness functions

The goal of the optimisation is to identify subnetworks that are differentially expressed and that define highly interconnected network modules. We first assign a z-score to a subnetwork S defining it by

(1) $$ z_{N}^{(S)} = \frac{1}{\sqrt{| N |}} \sum_{i\in N} | z_{i} |,  $$

where *z*_*i*_ is the z score of node *i*, *N* is the set of nodes in the subnetwork, and |*N*| its size [5, 8]. We then define two fitness functions accounting for the cases in which optimisation aims at finding one differentially expressed network module under two conditions or two different communities. In the first case the fitness function is defined by

(2) $$ F_{\cap} = \left| z_{N}^{(S_{1})}\right| + \left| z_{N}^{(S_{2})}\right| + \langle \mathbf{C} \rangle  $$

where $z_{N}^{(S_{1})}$, $z_{N}^{(S_{2})}$ are the z-scores of the subnetworks *S*_1_, *S*_2_ and 〈**C**〉 is the average clustering coefficient. In the second case it is defined by

(3) $$ F_{\cup} = \left| z_{N}^{(S_{1})}\right| + \left| z_{N}^{(S_{2})}\right| + Q  $$

where *Q* is the modularity of the network given by *S*_1_∪*S*_2_ [7]. When the modules are two, *Q* is defined by

(4) $$ Q = \frac{1}{2E} \left(\sum_{i,j \in N} \left[ A^{(S_{1} \cup S_{2})}_{ij} - R_{ij}\right] \right),\qquad Q\in\, [\!-1,1]  $$

where $A^{(S_{1} \cup S_{2})}_{\textit {ij}}$ is the adjacency matrix of the subnetwork *S*_1_∪*S*_2_ with *E* edges and the matrix **R**=(*R*_*ij*_) defines the null model against which the network is compared. The matrix element *R*_*i*,*j*_ is given by

$$R_{i,j} = \frac{k_{i} k_{j}}{2E} $$

where $k_{i} = \sum _{j} A^{(S_{1} \cup S_{2})}_{\textit {ij}}$ is the degree of node *i*. The objective function is then maximised by minimising the function

$$\bar{F} = \frac{1}{1+F},\qquad \text{with \(F=F_{\cap}~ \text{or}~ F_{\cup}\)}. $$

#### Mutation

The mutation operator iteratively selects a random node of an individual and verifies if removal of this node maintains the connection of the remaining network by applying a depth first search algorithm. If such a node has been identified within a fixed number of iterations, this node is removed and it is replaced with a nearest neighbour of another randomly selected node. When the algorithm is set to search for two different differentially expressed communities, node removal and substitution occurs in each of the two disjoint sets of nodes.

#### Crossover

The crossover operator is active only when two individuals share a common node. In such case the two sets of nodes are merged to define a connected network. Two nodes are then randomly sampled within this network and two new individuals are initialised by applying a depth first search algorithm. Similarly to what was defined for the mutation operator, when the algorithm is set to search for two separate differentially expressed communities, the two new individuals are selected to maintain the same number of nodes associated with each community.

#### Initialisation

The algorithm is initialised to search either for one community which is differentially expressed under two conditions or for two different communities each differentially expressed under a condition. In order to guarantee that each individual is sampled as a single connected component, initialisation is performed by randomly selecting one node of the network and applying a depth first search algorithm starting from this node. The algorithm is stopped when the search reaches the predefined size. When the algorithm is set to search for two different differentially expressed communities, each individual is sampled in order to be composed of a network comprising two disjoint sets of nodes, each defining a single connected component. A C implementation of our algorithm is reported in Additional file 1.

## Results and discussion

In what follows we firstly describe how the experimental and synthetic data were pre-processed, we then present the evaluation of the performance of our optimisation algorithm on synthetic data and finally an application to the experimental data set.

### Data pre-processing

#### Experimental data

Microarray data were downloaded from the NCBI Gene Expression Omnibus website [16] and normalised using the GEO2R R script [17].

These data were obtained by using high-density oligonucleotide microarrays that interrogate 10,000 full-length genes to compare gene expression patterns in CD, UC and a third non-IBD colitis group. Endoscopic biopsies of inflamed and uninflamed intestinal tissue from patients with IBD or controls were obtained from various regions of the colon whose sites of biopsy were categorised as sigmoid, transverse, ascending, descending colon; splenic flexure; hepatic flexure. The samples were labelled as ‘affected’, when taken from an area that appeared grossly affected (inflamed), or as ‘unaffected’, when taken from an area that appeared disease free (uninflamed) and was 10 cm from diseased areas. The dataset includes a total of 36 expression profiles from 4 colonoscopic biopsies from normal adults, 7 from adults with inflamed colon with CD, 12 from adults with non-inflamed colon with CD, 5 from adults with inflamed colon with UC, 4 from adults with non-inflamed colon with UC, 2 from adults with inflamed colon with bacterial infectious colitis, 1 from an adult with inflamed colon with indeterminate colitis, 1 from an adult with non-inflamed colon with indeterminate colitis. In our analysis we only considered expression profiles derived from CD patients, UC patients and healthy controls. Differentially regulated genes were selected as follows.

The Benjamini and Hochberg false discovery rate method was selected by default to adjust *p*-values for multiple testing. We selected as differentially expressed genes those whose *p*-value was minor than 0.05, log2 mean expression index was greater than 6.64 and logarithmic fold change was greater than 1. The threshold for the log2 mean expression index was selected following the threshold chosen by Wu et al. [4], this threshold being higher than the log2 mean expression in the microarray data (mean =6.5).

The interactome was obtained from iRefWeb [18], a web interface to protein interaction data consolidated from 10 public databases (BIND, BioGRID, CORUM, DIP, IntAct, HPRD, MINT, MPact, MPPI and OPHID). Two networks associated with inflamed and uninflamed tissues were built by selecting all interactions containing at least one differentially regulated node and such that nodes that are not differentially regulated act as link between two differentially regulated nodes; this enables inclusion of indirect interactions, as suggested by Rossin et al. [19].

The inflamed network comprised 666 interactions and 312 nodes of which 105 were differentially expressed in at least one condition; the uninflamed network included 645 interactions and 304 nodes of which 74 were differentially expressed in at least one condition.

These two networks include a single connected component with average degree approximately equal to 4.2 (Additional file 2: Figures S1 and S2 and Additional file 3). Following Ideker et al. [5], Z-scores of differentially expressed nodes were evaluated from their corresponding *p*-value, calculated under each condition, whereas the other nodes were given the zero value.

#### Synthetic data

**Fig. 2:**
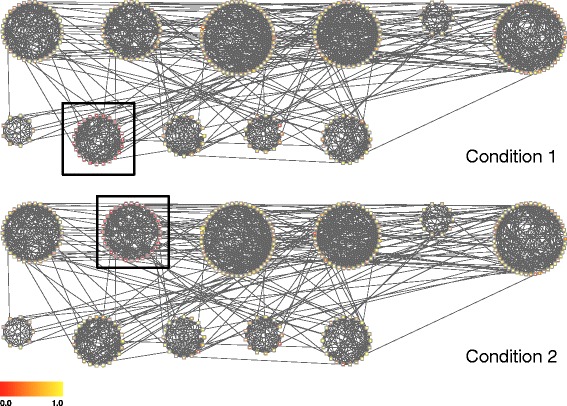
Synthetic data set. A simulated network with simulated differential expression in network communities under two conditions. Differentially expressed communities are highlighted within a rectangular box; node colours represent simulated p-values

Simulated networks were generated by applying the benchmark proposed by Lancichinetti et al. [20]. This benchmark extends the one proposed by Girvan and Newman [21] accounting for the distributions of node degrees and community size; both degree and community size distributions are assumed to be power laws and the modularity of the community structure depends on a mixing parameter *γ* specifying the fraction of links that each node shares with other nodes that are not part of its community. Simulated gene expression data were generated as follows. Firstly, we selected the first community size among the ones of size *s*_*c*1_, where *s*_*c*1_ is the largest size which is smaller or equal than the average of all the community sizes. We then selected the second community size *s*_*c*2_ either to be the closest bigger, when we simulated two different differentially expressed communities, or to be the same community, when we assumed that the same community was differentially expressed under both conditions. Simulated z-scores were then generated as follows

(5) $$ {\selectfont{\begin{aligned} {}z_{i} \sim \left\{ \begin{array}{rl} \pm \mathcal{N}(\mu_{1},\sigma) & \text{if \textit{i} is differentially expressed},\\ \mathcal{N}(\mu_{2},\sigma) & \text{otherwise} \end{array} \right. \quad i=1,\ldots,N \end{aligned}}}  $$

where *N*=300 is the number of nodes, *μ*_1_=3, *μ*_2_=0 and the sign is randomly chosen. Networks were generated by varying the parameters *γ* and *σ* within the range 0.1,0.2,…,0.5. When varying one parameter the other was maintained at the fixed value 0.1. In addition, four networks were generated for each parameter choice with average degree given by 〈*k*〉=4,6,8,10. All of the networks obtained were composed of a single connected component. A representation of a simulated network is presented in Fig. 2.

### Optimisation of synthetic networks

In order to verify the performance of our algorithm we generated a synthetic data set so that the solution found can be compared with a known optimal solution. We calculated two performance metrics: the prediction accuracy (PA) and the normalised mutual information (NMI). The first metric was applied to evaluate the optimisation performance when searching for differentially regulated subnetworks [8], the second one was shown to be appropriate for network partitioning [13, 22]. The prediction accuracy of an optimised subnetwork *S*_*O*_, compared with an actual subnetwork *S*_*A*_, is given by

$$PA(S_{O}, S_{A}) = 1 - \frac{FN + FP}{TN + TP}, $$

where *FN*, *FP*, *TN*, *TP* denote the number of false negatives, false positives, true negatives and true positives respectively.

We evaluated the average performance of 30 runs of our optimisation algorithm in each synthetic dataset using the parameters reported in Table 1.

**Table 1:** Algorithm parameters. Parameters used in all runs of our evolutionary algorithm

Parameter	Value
Number of generations	100
Population size	200
Crossover rate	0.1
Mutation rate	0.9

Under all perturbations the prediction accuracy was found to be larger than 0.8, showing higher performance in networks with lower average degree (see Additional file 2: Figure S3). In particular, this metric was larger than 0.9 when evaluated from networks with average degree approximately equal to the one of the experimental networks (〈*k*〉=4).

We then evaluated the performance of the same optimised subnetworks using the normalised mutual information. Denoting by *P*_*O*_, *P*_*A*_ the partitions defined by the subnetworks *S*_*O*_, *S*_*A*_ respectively, the normalised mutual information is given by [13]

$${}NMI(P_{O}, P_{A}) = \frac{-2 \sum_{i=1}^{c_{P_{O}}} \sum_{j=1}^{c_{P_{A}}} C_{ij} \log{\left(\frac{C_{ij} N}{C_{i \cdot} C_{\cdot j}} \right)} }{ \sum_{i=1}^{c_{P_{O}}} C_{i \cdot} \log{\left(\frac{C_{i \cdot}}{N} \right)} + \sum_{j=1}^{c_{P_{A}}} C_{\cdot j} \log{\left(\frac{C_{\cdot j}}{N} \right)} } $$

where *C* is the confusion matrix; $c_{P_{O}}$, $c_{P_{A}}$ are the number of groups in the partitions *P*_*O*_, *P*_*A*_; *C*_*i*·_, *C*_·*j*_ are the sum of elements of *C* in row *i* and column *j* and *N* is the number of nodes. Networks with average degree 〈*k*〉=4 presented a normalised mutual information approximately equal to 1 when varying the parameter *σ*, whereas they showed a decrease in performance when *γ* approached the value at which the community structure is lost, *γ*=0.5 (see Additional file 2: Figure S4).

### Optimisation of CD and UC networks

After having evaluated the performance of our evolutionary algorithm on synthetic data sets, we applied it to the experimental data set for the purpose of identifying dys-regulated modules in CD and UC. We then analysed the optimisation results by varying sub-network size and identified enriched pathways and biological processes under different conditions, these being inflamed and uninflamed tissues in CD and UC patients. We ran our algorithm by varying subnetworks sizes within the range 10,15,…,40 with 30 runs per size. All of the optimal sub-networks found had statistically significant z-scores relatively to their corresponding condition (|*z*|>5.8, *p*-value <6.10^−7^) confirming their association with disease.

**Fig. 3:**
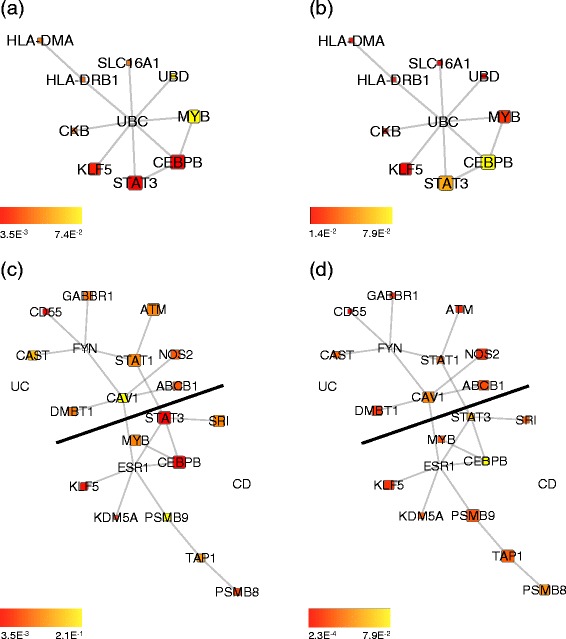
Best solutions found in affected tissues (size **1** **0**). Best subnetworks found within the results of 30 optimisation runs among subnetworks of size 10 and with differential expression evaluated from biopsies taken from affected tissues. (**a**), (**b**) Overlapping network module which is differentially expressed both in CD and UC. (**c**), (**d**) Non-overlapping network modules that are differentially expressed either in CD or in UC; the black line indicates the boundary between nodes associated with CD and nodes associated with UC. Node colours are proportional to the node *p*-value in CD (**a**), (**c**) and UC (**b**), (**d**). Node size is proportional to its identification frequency when applying our evolutionary algorithm by varying network size (see section Results and discussion)

**Fig. 4:**
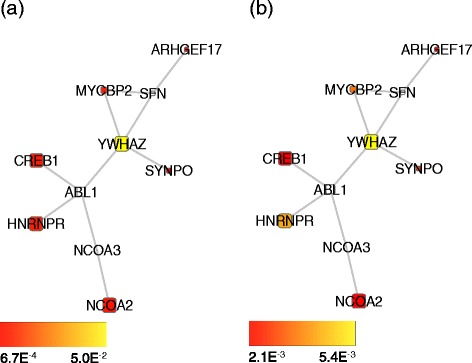
Best overlapping module found in unaffected tissues (size **1** **0**). Best overlapping module found after 30 optimisation runs among subnetworks of size 10 and with differential expression evaluated on unaffected tissues; the module is differentially expressed both in CD and UC. Node colour is proportional to the node *p*-value in CD (**a**) and UC (**b**). Node size is proportional to its identification frequency when applying our evolutionary algorithm by varying network size (see section Results and discussion in the main text)

**Fig. 5:**
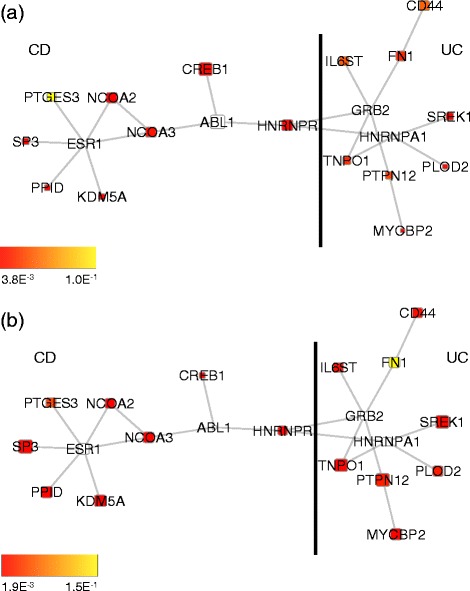
Best non-overlapping modules found in unaffected tissues (size **1** **0**). Best non-overlapping modules found after 30 optimisation runs among subnetworks of size 10 and with differential expression evaluated on unaffected tissues. The modules are differentially expressed either in CD or UC. The black line indicates the boundary between nodes associated with CD and nodes associated with UC. Node colour is proportional to the node *p*-value in CD (**a**) and UC (**b**). Node size is proportional to its identification frequency when applying our evolutionary algorithm by varying network size (see section Results and discussion in the main text)

As exemplar solutions, we report the best subnetworks found of size 10 in Figs. 3, 4 and 5.

The algorithm enabled the identification of subnetworks which are differentially expressed both in CD and UC (see Figs. 3a,b and 4a,b) and of connected pairs of subnetworks, each composed of 10 nodes, forming a differentially expressed subnetwork in CD biopsies and a differentially expressed subnetwork in UC biopsies (see Figs. 3c,d and 5a,b). We then wondered whether we could highlight a particular subnetwork size by analysing its functional homogeneity. To this end, for each sub-network found, we calculated a functional similarity score to examine if, within this range, there was a clear optimal size in terms of similarity in biological processes (see Additional file 2: Figures S5 and S6) [7, 23]. Since no such particular size was identified, we then evaluated the frequency of occurrence of each node in the optimal solutions when varying sub-network size. Fixing a frequency threshold >0.3 and mapping the selected nodes on the interaction network, we derived the subnetworks whose largest connected components are depicted in Additional file 2: Figures S7 and S8.

Such networks show several overlapping and non-overlapping nodes in CD and UC patients and small overlap among the most frequently identified nodes between inflamed and uninflamed tissues (see Additional file 3).

The solutions found highlight cross-talk among enriched pathways, mainly among the JAK/STAT signalling pathway, EGF receptor signalling pathway, Gonadotropin releasing hormone receptor pathway and p38 MAPK pathway (see Additional file 2: Figures S9, S10 and S11). The EGF receptor signalling pathway acts by phosphorylating the Janus kinases (JAK) resulting in the activation of Signal Transducer and Activator of Transcription proteins (STATs) and plays a role in regulating inflammation, in particular during colitis [24, 25]. Although the exact role of STAT3 in the pathogenesis of CD is not understood, mice with tissue-specific disruption of Stat3 show CD-like pathogenesis and constitutively phosphorylated STAT3 is found in intestinal T cells from patients with CD. These results support the notion that dys-regulation of STAT3 signalling might be involved in fuelling inflammation in CD [26]. p38 is a member of the mitogen-activated protein kinase (MAPK) family, which is composed of ubiquitously expressed kinases playing important roles in various signal transduction pathways in mammalian cells [27–30].

We found that nodes in the averaged overlapping subnetwork in inflamed tissues were enriched in the JAK/STAT signalling pathway, whereas nodes in uninflamed tissues were mainly enriched in the EGF receptor signalling pathway, Gonadotropin releasing hormone receptor pathway and p38 MAPK pathway (see Additional file 2: Figure S9). Nodes in the averaged non-overlapping subnetworks associated with CD in inflamed tissues were enriched in the JAK/STAT and EGF receptor signalling pathway components, the same being true for nodes associated with UC (see Additional file 2: Figure S10). Nodes in the averaged non-overlapping subnetworks associated with CD in uninflamed tissues were mainly enriched in the EGF receptor signalling, Gonadotropin releasing hormone receptor and p38 MAPK pathway, whereas no enriched pathways were found comprising nodes associated with UC (see Additional file 2: Figure S11). Enrichment in biological processes highlighted involvement of several metabolic, developmental and cell communication processes in the networks above mentioned (see Additional file 2: Figures S12, S13 and S14). From the network topology viewpoint, the subnetworks selected comprise several hubs and hidden nodes, these are reported in Additional file 3 together with the list of subnetwork nodes.

In order to compare the results of our method with existing methods for gene set enrichment, we tested the algorithm Gene Set Enrichment Analysis (GSEA) on the CD-UC microarray data set [31]. GSEA is a computational method that determines whether an a priori defined set of genes shows statistically significant, concordant differences between two biological states (e.g. phenotypes) and enables the identification of core members of high scoring gene sets that contribute to the enrichment score (Leading-Edge Subset). GSEA may not identify dysregulated subnetworks and communities but it may identify dysregulated sets of genes that can be compared with the subnetworks optimised with our algorithm. The gene set database was obtained from the Molecular Signatures Database (MSigDB), which is a collection of annotated gene sets for use with the GSEA software, and includes gene sets that represent cell states and perturbations within the immune system [32]. We ran GSEA on four phenotypes: inflamed tissues in CD versus control, inflamed tissues in UC versus control, uninflamed tissues in CD versus control and uninflamed tissues in UC versus control. We extracted the leading edge subsets for gene sets with FDR q-val <0.01. STAT1, STAT3 and JAK2 were included in the leading edges obtained from inflamed tissues in CD, whereas STAT1, STAT3 were found in the leading edges obtained from inflamed tissues in UC. We then selected all genes in these leading edge subsets and analysed their over-representation in the nodes of our averaged networks: overlapping nodes in CD and UC in inflamed and uninflamed tissues, non-overlapping nodes in CD in inflamed and uninflamed tissues, non-overlapping nodes in UC in inflamed and uninflamed tissues (Fisher’s exact test). Five of six lists of network nodes were found to be significantly enriched (*p*-value <0.01) except for the list of non-overlapping nodes in UC in uninflamed tissues (see Additional file 2: Figure S15).

Some of the nodes identified by our optimisation algorithm have been identified in GWAS for CD and UC [33] (see Table 2); moreover, STAT3, NOS2, PSMB10 were prioritised by a previous network analysis based on GWAS data [34]. Defects in autophagy pathways have been implicated in Crohn’s pathogenesis and we found autophagy related genes in our optimised subnetworks: AKAP9, AKT1, ATM, BNIP3L and NCOA2 (Autophagy Database [35]) (see Table 3). Accumulating data suggest mitochondria as integrators of autophagy and inflammation signalling pathways; consequently it is possible that mitochondrial stress participates in the pathology of IBD [36]. The genes BAD and TAP1, identified by our optimisation, are reported in the MITOCARTA database, which collects 1013 genes encoding proteins with strong support of mitochondrial localisation based on homology to mouse MitoCarta genes [37] (see Table 3). Other identified genes are related to the MHC class I antigen processing pathway, namely PSMB9, PSMB10 and TAP1 [4] which is interesting given the recent identification of CD8 T cell signatures linked to inflammation in Crohn’s [38]. Some of the genes discussed above and identified by our subnetworks were not identified by the GSEA Leading-Edge Subsets for the corresponding experimental condition, these being the GWAS reported gene GABBR1 and the Mitocarta and 33 genes AKAP9, AKT1, BAD; in addition, NCOA2 was also not identified by GSEA in affected tissues. Notably AKT1 (autophagy related) and BAD (mitochondrial localisation) act as hidden nodes that, although not being differentially expressed, play a role in connecting differentially expressed genes (see Additional file 2: Figure S8c).

**Table 2:** GWAS genes

Genes	GWAS	Subnetworks
STAT3	CD	OV_A, CD_A
GABBR1	CD	OV_A, CD_A
PSMB9	CD	OV_A, CD_A
NOS2	CD	OV_A, CD_A
PSMB10	CD	CD_A
IL7R	UC	OV_A
IL6ST	CD	OV_U, CD_U

Genes identified by our optimisation algorithm and reported in GWAS studies in CD and UC. CD_A: CD affected (inflamed), UC_A: UC affected (inflamed), OV_A: (overlap) CD and UC affected (inflamed), CD_U: CD unaffected (uninflamed), UC_U: UC unaffected (uninflamed), OV_U: (overlap) CD and UC unaffected (uninflamed)

**Table 3:** Mitocarta and autophagy genes

Genes	Autophagy	Mitocarta	Subnetworks
AKAP9	Y	N	CD_A
AKT1	Y	N	OV_U, CD_U
ATM	Y	N	OV_A, CD_A, UC_A
BAD	N	Y	CD_U
BNIP3L	Y	N	OV_U, CD_U
NCOA2	Y	N	OV_A, CD_A, UC_A, OV_U, CD_U, UC_U
TAP1	N	Y	OV_A, CD_A, UC_A

Genes identified by our optimisation algorithm and reported in the Mitocarta and Autophagy databases [35, 37]. CD_A: CD affected (inflamed), UC_A: UC affected (inflamed), OV_A: (overlap) CD and UC affected (inflamed), CD_U: CD unaffected (uninflamed), UC_U: UC unaffected (uninflamed), OV_U: (overlap) CD and UC unaffected (uninflamed)

## Conclusions

The availability of large scale interactome data enables unbiased analysis of gene expression data from a network perspective. Optimisation algorithms aimed at identifying differentially expressed network modules may help to highlight interactions among known molecular pathways not yet reported in pathway databases. Because of the computational complexity of such an optimisation problem, stochastic algorithms have been suggested as useful approaches to extract such information [5, 8]; in particular, evolutionary algorithms are a suitable choice for this purpose since they are able to identify close to optimal solutions in fitness functions with several local minima [8].

We have proposed an evolutionary algorithm to identify dys-regulated network modules in microarray data derived under two disease conditions. The algorithm integrates a molecular interaction network with gene expression data and optimises differentially expressed network modules accounting for community structure. The algorithm performance was first evaluated on synthetic data sets resembling the topological structure of networks reported in biological databases and it was then applied to an experimental dataset comprising a human interactome and microarray data generated from biopsies in patients with CD and UC [4]. Optimisation was performed by varying the subnetwork size and differential expression of the identified subnetworks was found to be statistically significant in all of the evaluated sizes. Analysis of occurrence of the nodes identified by varying network size showed that the most frequently identified nodes comprised network hubs and hidden nodes whose role is maintenance of network connectivity. The solutions found highlighted cross-talk among enriched pathways and the nodes identified may warrant biological investigation.

## Additional files

Additional file 1
**Code.zip — Folder including the C code used in our optimisation.** (ZIP 727 kb)

Additional file 2
**Supporting_Information.pdf — PDF document including supplementary.** Text and figures as referred to in the main text. (PDF 1458 kb)

Additional file 3
**Supplementary_Workbook.xls — Excel workbook comprising the interaction networks described in the main text and in the Supporting Information.** Legends of each workbook sheet are included in the workbook ‘Summary’ sheet. (XLSX 210 kb)

## References

[1] Jostins L, Ripke S, Weersma RK, Duerr RH, McGovern DP, Hui KY (2012). Host-microbe interactions have shaped the genetic architecture of inflammatory bowel disease. Nature.

[2] Cho DY, Kim YA, Przytycka TM. Chapter 5: Network Biology Approach to Complex Diseases. PLoS Comput Biol. 2012;8(12). e1002820. 10.1371/journal.pcbi.1002820 PMC3531284 23300411

[3] Kim YA, Przytycka TM (2013). Bridging the Gap between Genotype and Phenotype via Network Approaches. Front Genet.

[4] Wu F, Dassopoulos T, Cope L, Maitra A, Brant SR, Harris ML (2007). Genome-wide gene expression differences in Crohn’s disease and ulcerative colitis from endoscopic pinch biopsies: insights into distinctive pathogenesis. Inflamm Bowel Dis.

[5] Ideker T, Ozier O, Schwikowski B, Siegel AF. Discovering regulatory and signalling circuits in molecular interaction networks. Bioinformatics. 2002;18. Suppl 1:S233–40. 10.1093/bioinformatics/18.suppl_1.s233 12169552

[6] Huang SS, Fraenkel E (2009). Integrating proteomic, transcriptional, and interactome data reveals hidden components of signaling and regulatory networks. Sci Signal.

[7] Lewis AC, Jones NS, Porter MA, Deane CM (2010). The function of communities in protein interaction networks at multiple scales. BMC Syst Biol.

[8] Klammer M, Godl K, Tebbe A, Schaab C (2010). Identifying differentially regulated subnetworks from phosphoproteomic data. BMC Bioinformatics.

[9] Goldberg DE. Genetic algorithms in search, optimization and machine learning. Upper Saddle River: Addison-Wesley; 1989.

[10] Eiben AE, Schoenauer M (2002). Evolutionary computing. Information Processing Letters.

[11] Chuang HY, Lee E, Liu YT, Lee D, Ideker T (2007). Network-based classification of breast cancer metastasis. Mol Syst Biol.

[12] Amiri B, Hossain L, Crawford J (2012). A hybrid evolutionary algorithm based on HSA and CLS for multi-objective community detection in complex networks. Proceedings of the 2012 IEEE/ACM International Conference on Advances in Social Networks Analysis and Mining ASONAM.

[13] Pizzuti C (2012). A multiobjective genetic algorithm to find communities in complex networks. IEEE T Evolut Comput.

[14] Gong M, Ma L, Zhang Q, Jiao L (2012). Community detection in networks by using multiobjective evolutionary algorithm with decomposition. Physica A: Statistical Mechanics and its Applications.

[15] GA: software developed at Kanpur Genetic Algorithms Laboratory. http://www.iitk.ac.in/kangal/codes.shtml. Accessed 15 November 2001.

[16] NCBI Gene Expression Omnibus - GSE6731. http://www.ncbi.nlm.nih.gov/geo/.

[17] GEO, 2R R script. http://www.ncbi.nlm.nih.gov/geo/geo2r.

[18] Turner B, Razick S, Turinsky AL, Vlasblom J, Crowdy EK, Cho E, et al. iRefWeb: interactive analysis of consolidated protein interaction data and their supporting evidence. Database. 2010:baq023. http://wodaklab.org/iRefWeb. Accessed 19 September 2013. 10.1093/database/baq023 PMC2963317 20940177

[19] Rossin EJ, Lage K, Raychaudhuri S, Xavier RJ, Tatar D, Benita Y, et al. Proteins Encoded in Genomic Regions Associated with Immune-Mediated Disease Physically Interact and Suggest Underlying Biology. PLoS Genet. 2011; 7(1). e1001273. 10.1371/journal.pgen.1001273 PMC3020935 21249183

[20] Lancichinetti A, Fortunato S, Radicchi F (2008). Benchmark graphs for testing community detection algorithms. Phys Rev E.

[21] Girvan M, Newman ME (2002). Community structure in social and biological networks. Proc Natl Acad Sci USA.

[22] Danon L, Diaz-Guilera A, Duch J, Arenas A. Comparing community structure identification. J Stat Mech. 2005:P09008.

[23] Pandey J, Koyutürk M, Subramaniam S, Grama A. (2008). Functional coherence in domain interaction networks. Bioinformatics.

[24] Dubé PE, Yan F, Punit S, Girish N, McElroy SJ, Washington MK (2012). Epidermal growth factor receptor inhibits colitis-associated cancer in mice. J Clin Invest.

[25] Andl CD, Mizushima T, Oyama K, Bowser M, Nakagawa H, Rustgi AK (2004). EGFR-induced cell migration is mediated predominantly by the JAK-STAT pathway in primary esophageal keratinocytes. Am J Physiol Gastrointest Liver Physiol.

[26] Shuai K, Liu B (2003). Regulation of JAK-STAT signalling in the immune system. Nat Rev Immunol.

[27] Chang L, Karin M (2001). Mammalian MAP kinase signalling cascades. Nature.

[28] Kyriakis JM, Avruch J (1996). Sounding the alarm: protein kinase cascades activated by stress and inflammation. J Biol Chem.

[29] Hollenbach E, Neumann M, Vieth M, Roessner A, Malfertheiner P, Naumann M (2004). Inhibition of p38 MAP kinase- and RICK/NF-kappaB-signaling suppresses inflammatory bowel disease. FASEB J.

[30] Waetzig GH, Seegert D, Rosenstiel P, Nikolaus S, Schreiber S (2002). p38 mitogen-activated protein kinase is activated and linked to TNF-alpha signaling in inflammatory bowel disease. J Immunol.

[31] Subramanian A, Tamayo P, Mootha VK, Mukherjee S, Ebert BL, Gillette MA, et al. Gene set enrichment analysis: a knowledge-based approach for interpreting genome-wide expression profiles. Proc Natl Acad Sci USA. 2005 Oct 25; 102(43):15545–50. 10.1073/pnas.0506580102 PMC1239896 16199517

[32] HIPC. The signatures were generated by manual curation of published studies in human and mouse immunology as part of the Human Immunology Project Consortium. http://www.immuneprofiling.org.

[33] Hindorff LA, MacArthur J, Morales J, Junkins HA, Hall PN, Klemm AK, et al. A Catalog of Published Genome-Wide Association Studies. Available: http://www.genome.gov/gwastudies. Accessed December 2014.

[34] Muraro D, Lauffenburger DA, Simmons A. Prioritisation and network analysis of Crohn’s disease susceptibility genes. PLoS One. 2014; 9(9). e108624. 10.1371/journal.pone.0108624 PMC4182533 25268122

[35] Autophagy Database. http://autophagy.info/autophagy/index.html.

[36] Rath E, Haller D. Mitochondria at the interface between danger signaling and metabolism: role of unfolded protein responses in chronic inflammation. Inflamm Bowel Dis. 2012 Jul; 18(7):1364–77. 10.1002/ibd.21944 22183876

[37] MITOCARTA database. http://www.broadinstitute.org/pubs/MitoCarta/human.mitocarta.html.

[38] Lee JC, Lyons PA, McKinney EF, Sowerby JM, Carr EJ, Bredin F (2011). Gene expression profiling of CD8+ T cells predicts prognosis in patients with Crohn disease and ulcerative colitis. J Clin Invest.

