# Supplementary material for: An integrative analysis of gene expression and molecular interaction data to identify dys-regulated sub-networks in inflammatory bowel disease
Source: BMC Bioinformatics. 2016 Jan 19;17:42. doi: 10.1186/s12859-016-0886-z (PMC4719745; doi:10.1186/s12859-016-0886-z)
Supplement: Additional file 2 — Supporting_Information.pdf — PDF document including supplementary. Text and figures as referred to in the main text. (PDF 1458 kb) [file 12859_2016_886_MOESM2_ESM.pdf]

Supporting Information

Supplementary Material

Functional similarity score

The functional similarity score is defined as follows [1], [2]. Given a sub-network S with N nodes the similarity measure in a molecular interaction network between gene i and j is defined by

$$G_{ij} = 1 - \frac{\log(n_{ij})}{\log N}$$

where n_{ij} is the number of genes sharing the same set of gene ontology terms and N is the total number of genes in the network. This score was evaluated on gene ontology terms with a threshold of minimal occurrence fixed to 10 and by varying the sub-network size in affected and unaffected tissues for overlapping subnetworks (see Supplementary Figure 5) and not-overlapping subnetworks (see Supplementary Figure 6).

Supplementary Figures

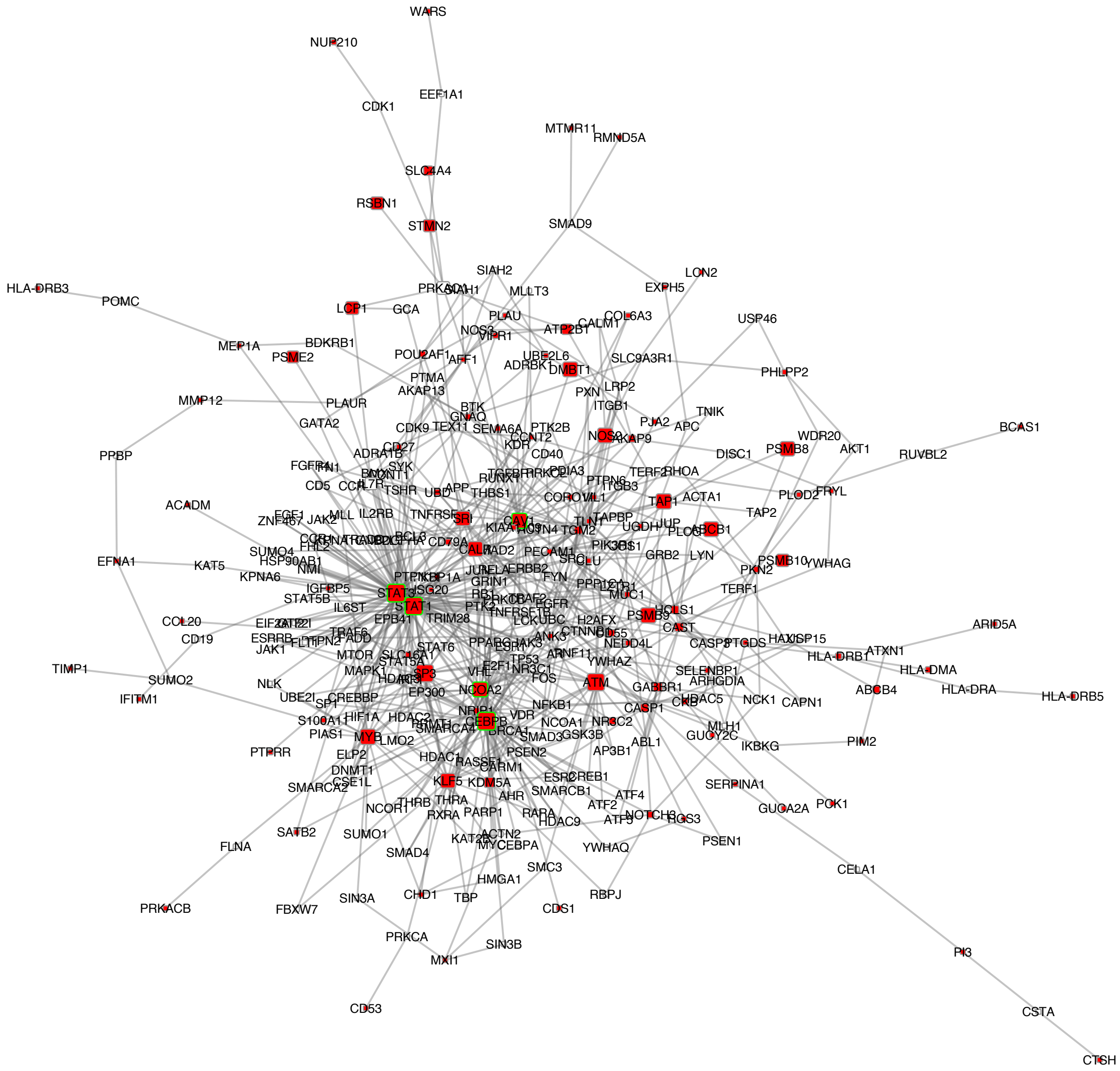

Figure 1: **Affected network.** Molecular interaction network derived from differentially expressed nodes in affected tissues and nearest neighbours of at least two differentially expressed nodes. Nodes differentially expressed either in CD or UC biopsies are highlighted in red. Node size is proportional to its identification frequency when applying our evolutionary algorithm by varying network size (see section Results and Discussion in the main text). The border of the first five hubs is highlighted in green.

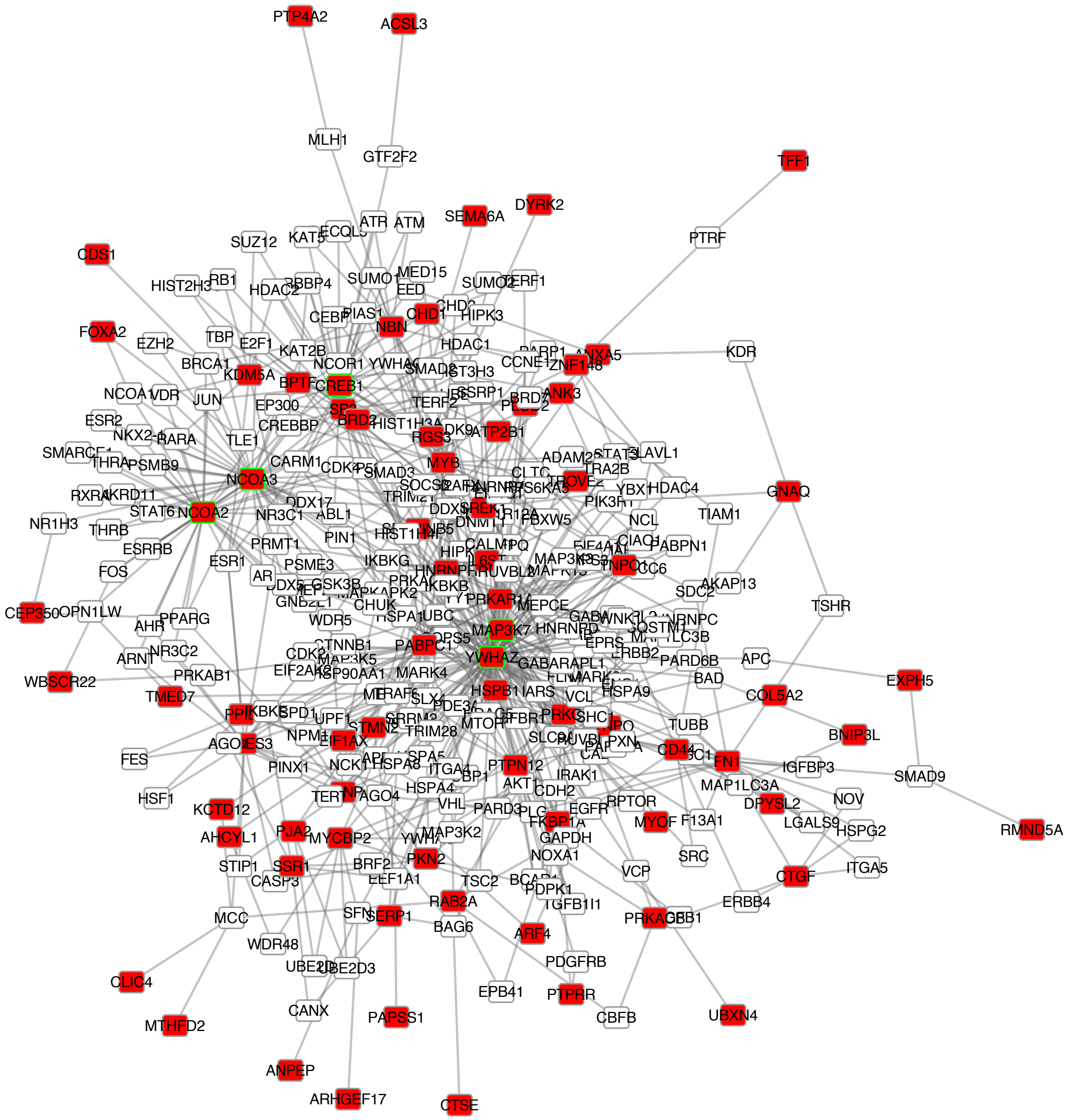

Figure 2: **Unaffected network.** Molecular interaction network derived from differentially expressed nodes in unaffected tissues and nearest neighbours of at least two differentially expressed nodes. Nodes differentially expressed either in CD or UC biopsies are highlighted in red. Node size is proportional to its identification frequency when applying our evolutionary algorithm by varying network size (see section Results and Discussion in the main text). The border of the first five hubs is highlighted in green.

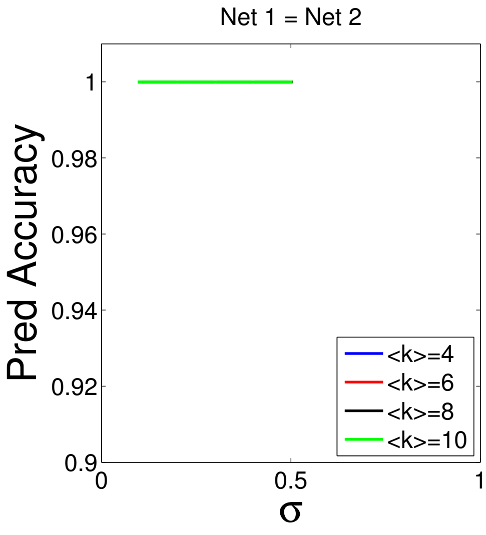

(a)

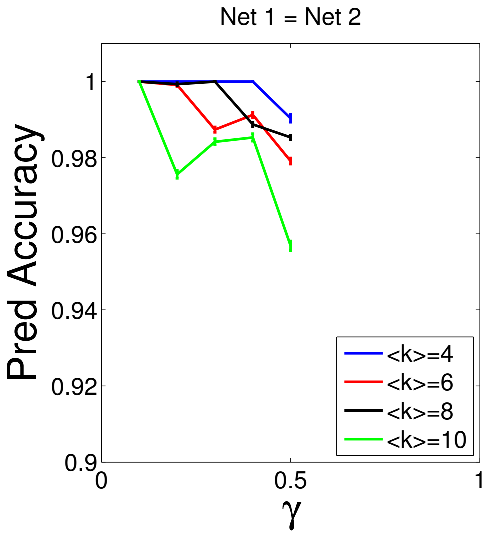

(b)

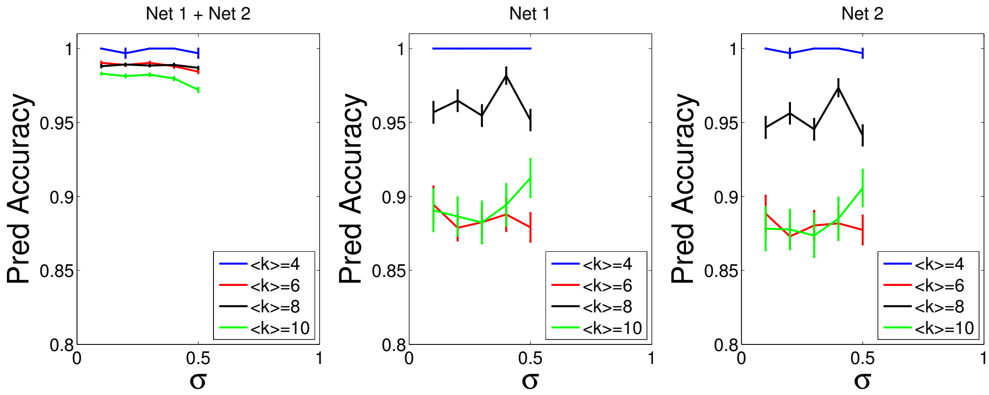

(c)

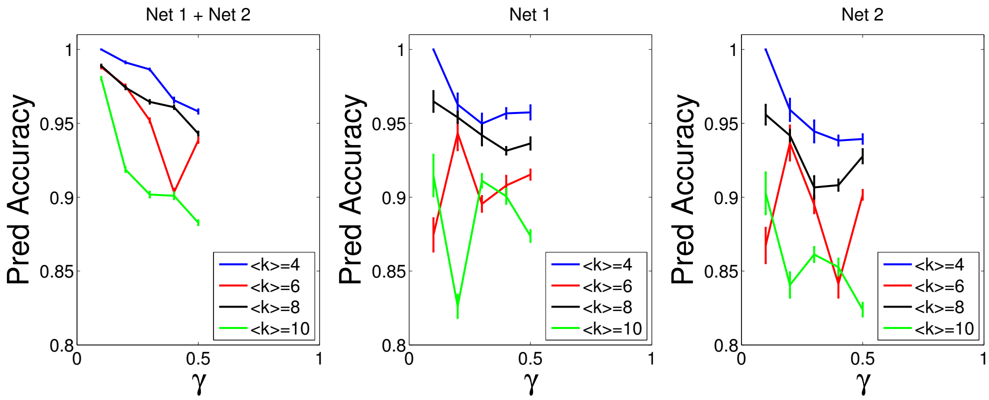

(d)

Figure 3: **Prediction accuracy.** Prediction accuracy of the synthetic data set when varying the parameters σ and γ in the following cases: (a),(b) the same community is differentially expressed under two conditions; (c),(d) two different communities are differentially expressed under each condition. For each choice of σ and γ, four networks were generated with average degrees $\langle k \rangle = 4, 6, 8, 10$. Mean values and standard deviations were calculated using the results of 30 optimisation runs.

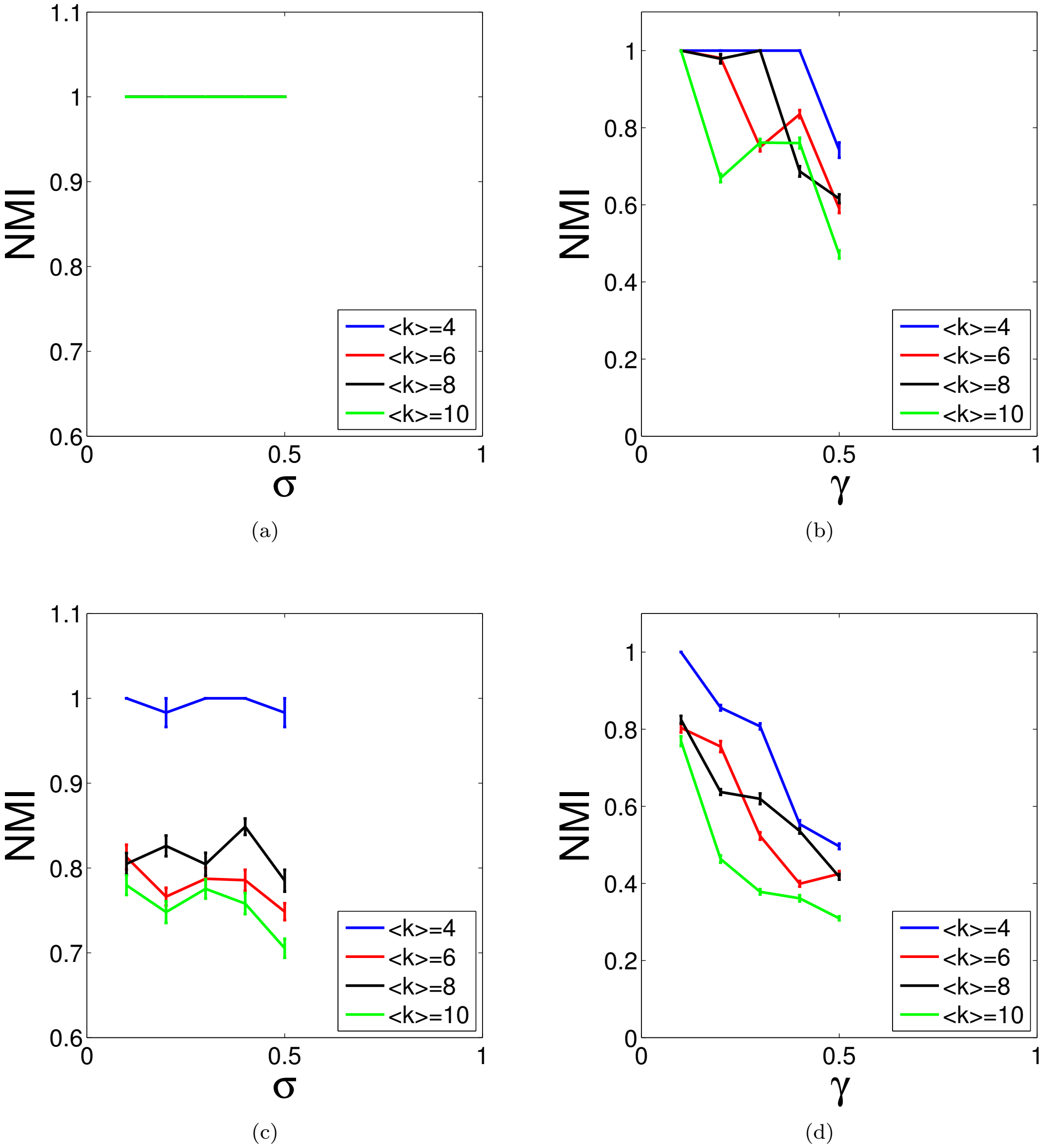

Figure 4: **Normalised Mutual Information (NMI).** NMI of the synthetic data set when varying the parameters σ and γ in the following cases: (a)-(b) the same community is differentially expressed under two conditions; (c)-(d) two different communities are differentially expressed under each condition. For each choice of σ and γ, four networks were generated with average degrees $\langle k \rangle = 4, 6, 8, 10$. Mean values and standard deviations were calculated using the results of 30 optimisation runs.

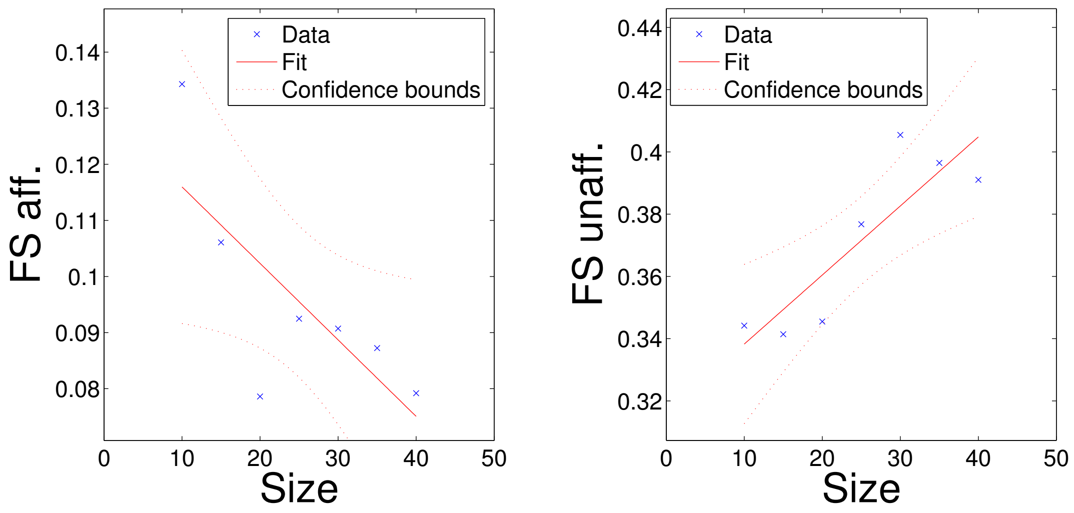

(a) (b)

Figure 5: **Functional Similarity score of overlapping sub-networks**. Functional Similarity score of optimal overlapping sub-networks found (mean of 30 optimisation runs) when varying the sub-network size in affected tissues (a) and unaffected tissues (b). F-test p-values of linear regression models < 0.05.

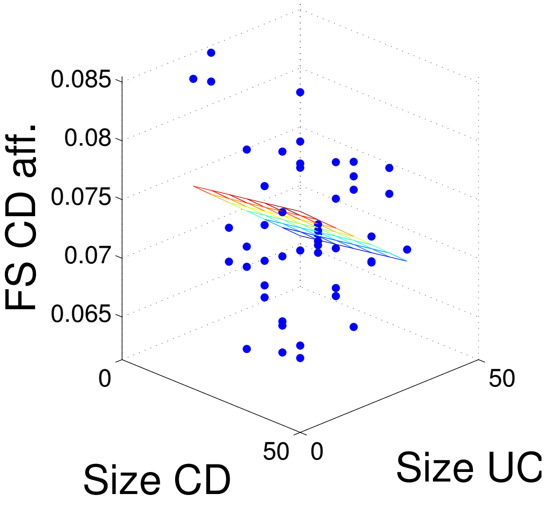

(a)

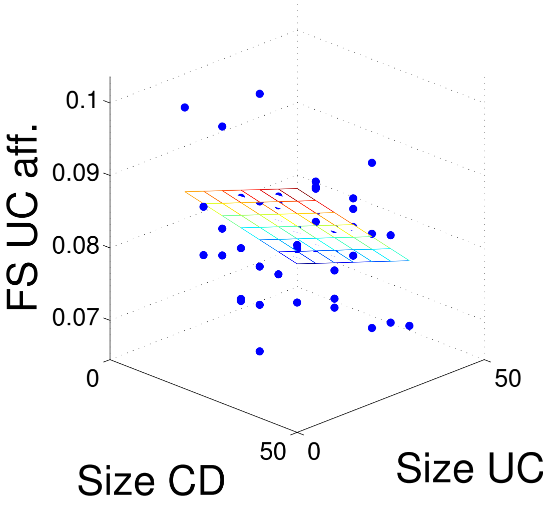

(b)

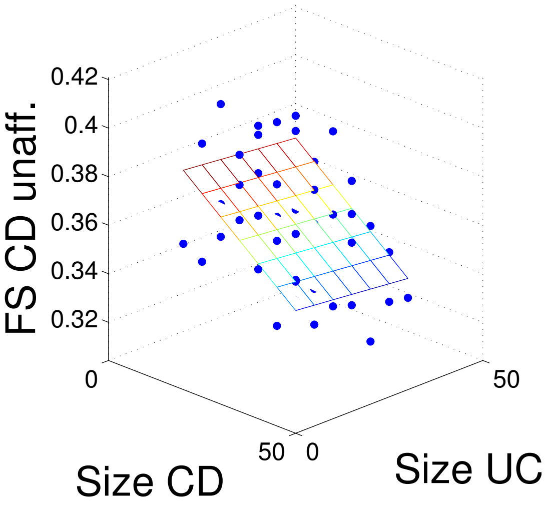

(c)

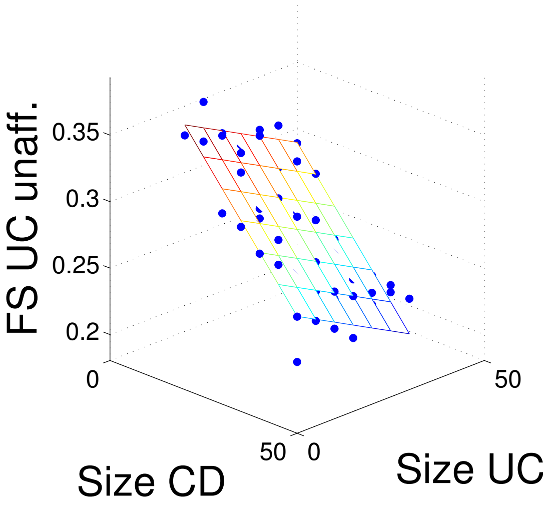

(d)

Figure 6: **Functional Similarity score of not-overlapping sub-networks**. Functional Similarity score of optimal not-overlapping sub-networks found (mean of 30 optimisation runs) when varying the sub-network size in affected tissues (a-b) and unaffected tissues (c-d). F-test p-values of linear regression models < 0.05.

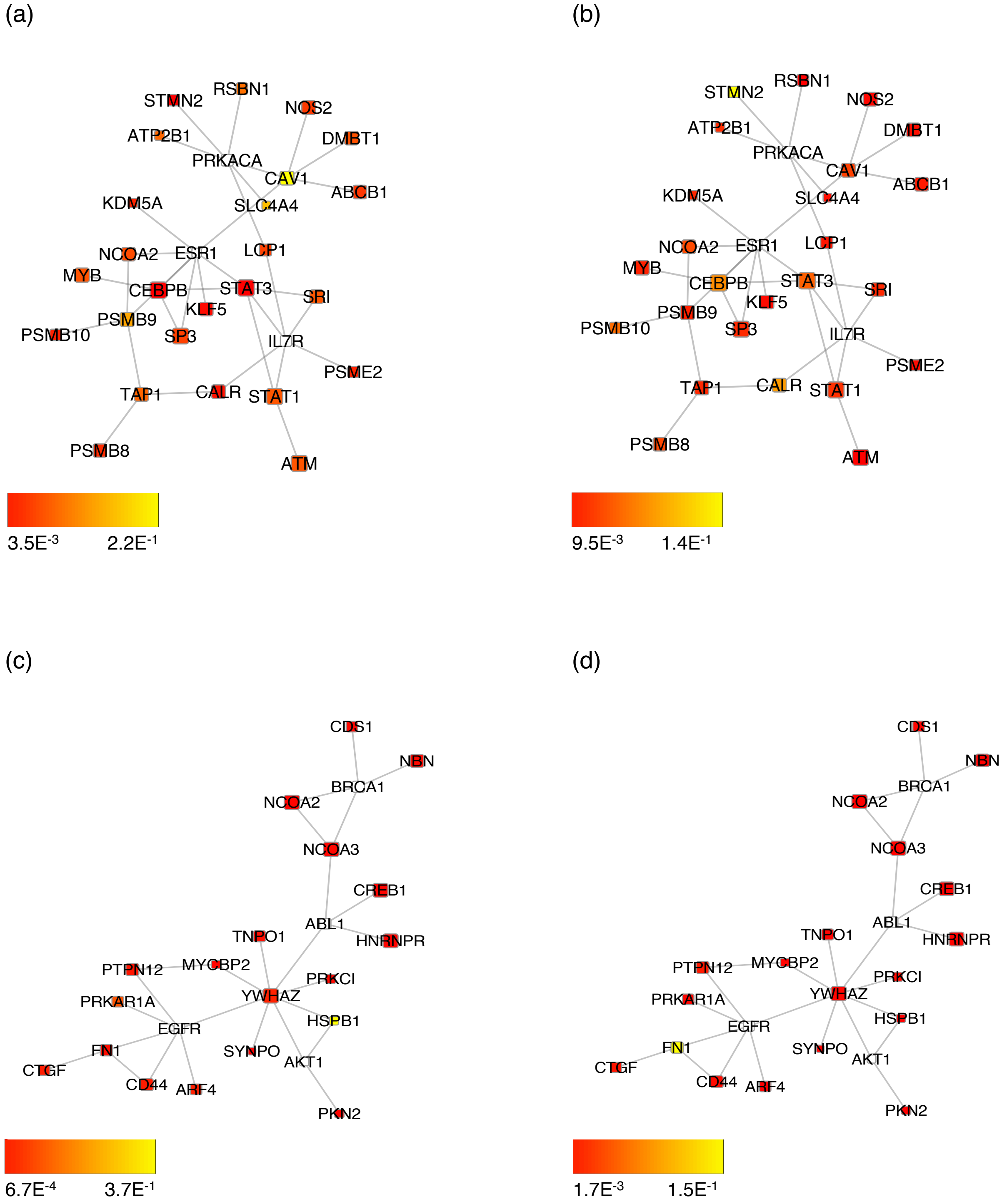

Figure 7: **Averaged network modules that are differentially expressed both in CD and UC.** Largest connected components of the subnetworks found when mapping into the interactome the nodes most frequently identified (frequency threshold > 0.3) by all the optimised overlapping modules (7 sizes $\times$ 30 runs $= 210$ subnetworks) in affected tissues (a), (b) and in unaffected tissues (c), (d). Node colours are proportional to the node p-value in CD (a), (c) and UC (b), (d). Node size is proportional to its identification frequency when applying our evolutionary algorithm by varying network size (see section Results and Discussion).

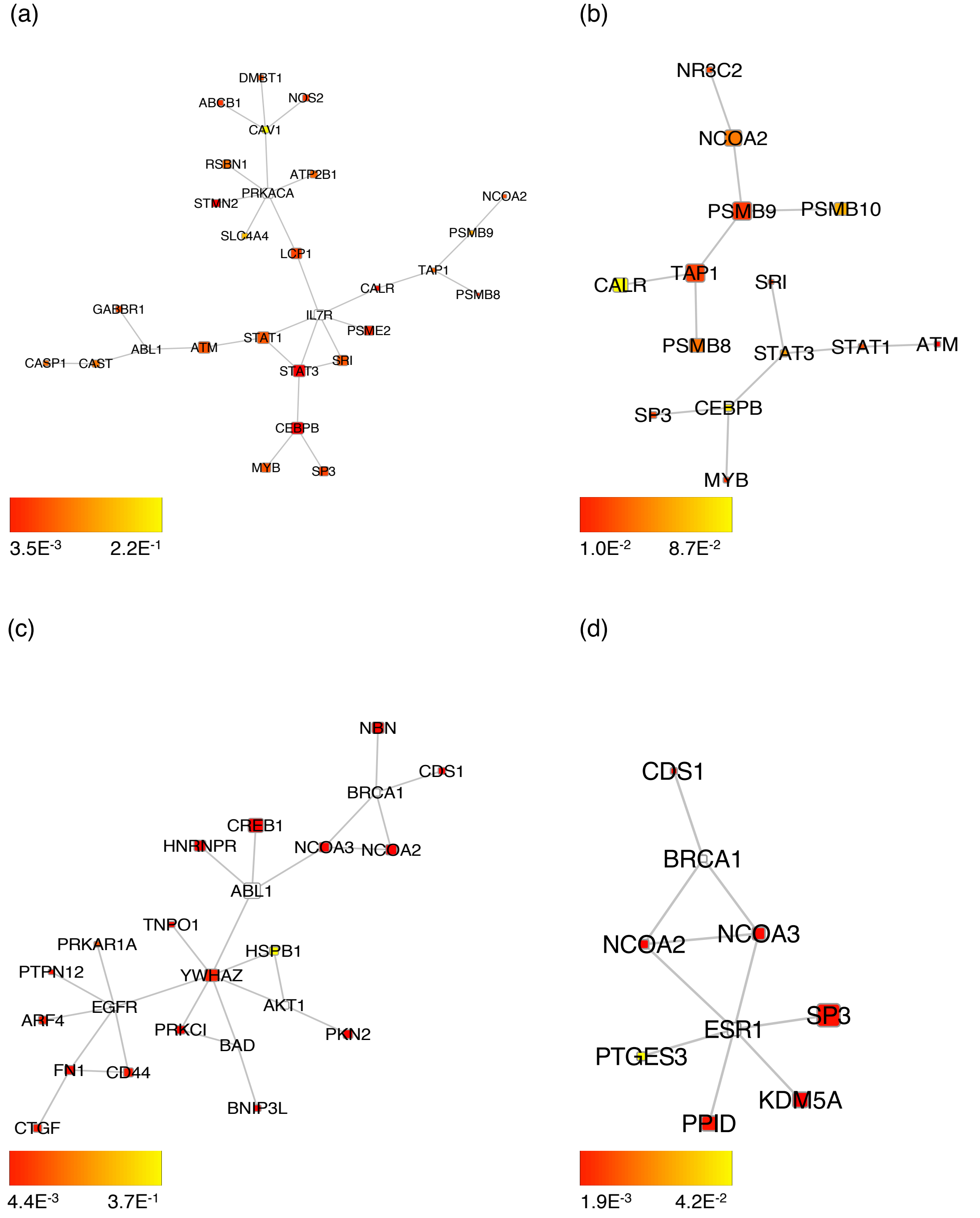

Figure 8: **Averaged network modules that are differentially expressed either in CD or UC.** Largest connected components of the subnetworks found when mapping into the interactome the nodes most frequently identified (frequency threshold > 0.3) by all the optimised non-overlapping modules (7 sizes CD $\times$ 7 sizes UC $\times$ 30 runs $=$ 1470 subnetworks) in affected tissues (a), (b) and in unaffected tissues (c), (d). Node colours are proportional to the node p-value in CD (a), (c) and UC (b), (d). Node size is proportional to its identification frequency when applying our evolutionary algorithm by varying network size (see section Results and Discussion).

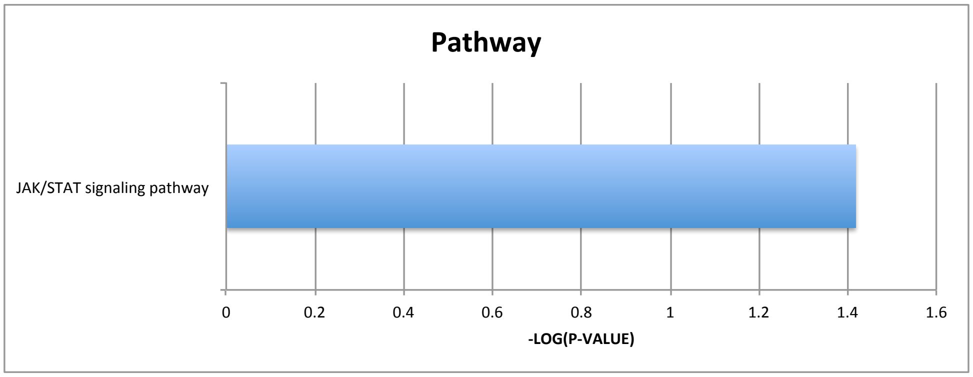

(a)

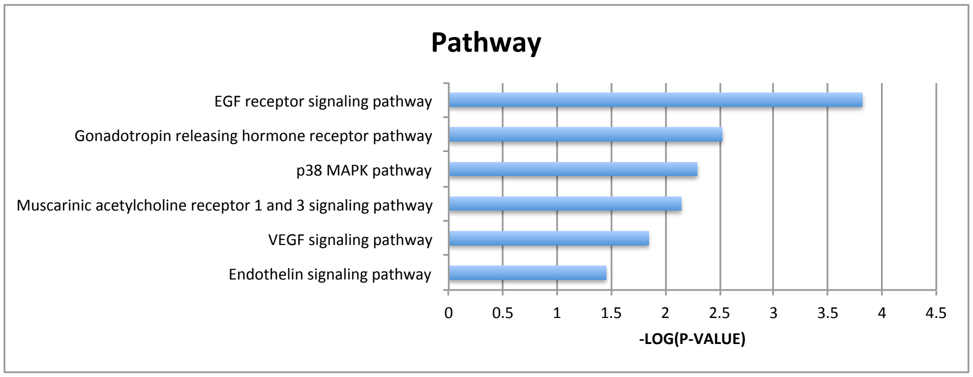

(b)

Figure 9: **Pathway enrichment in averaged modules that are differentially expressed both in CD and UC**. Chart summarising the pathways that are enriched in the nodes associated with CD and UC in affected (a) and unaffected (b) tissue in the averaged subnetworks that are differentially expressed both in CD and UC. P-value threshold was set to 0.05.

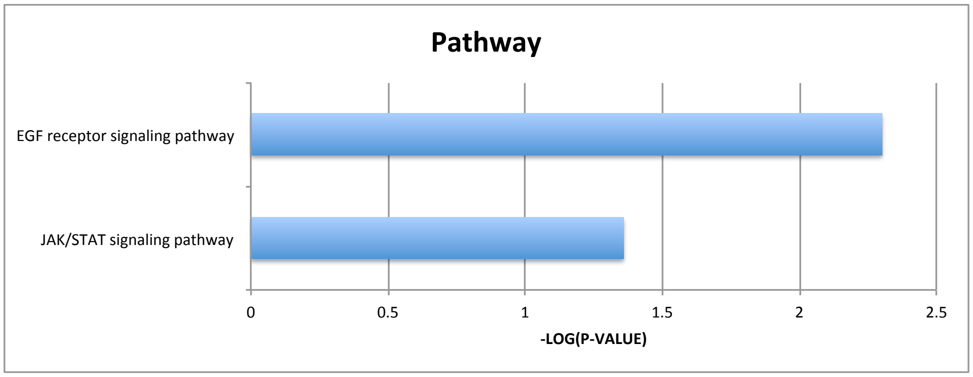

(a)

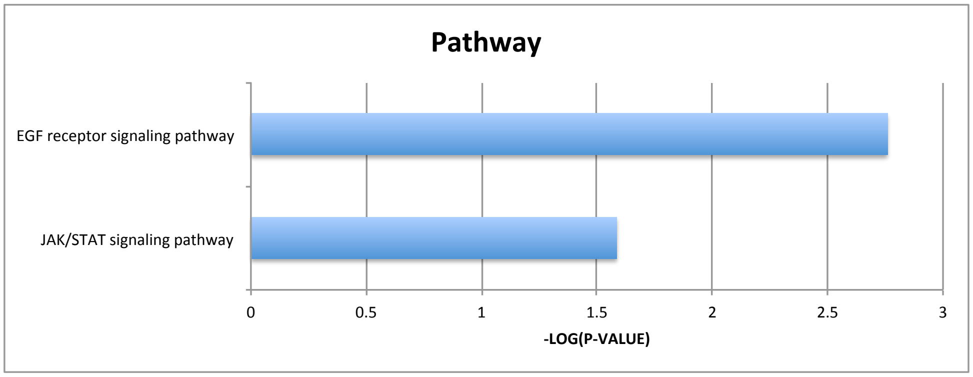

(b)

Figure 10: **Pathway enrichment in averaged modules that are differentially expressed either in CD or UC (affected tissues)**. Chart summarising the pathways that are enriched in the nodes associated with CD (a) and UC (b) in the averaged subnetworks that are differentially expressed either in CD or UC. P-value threshold was set to 0.05

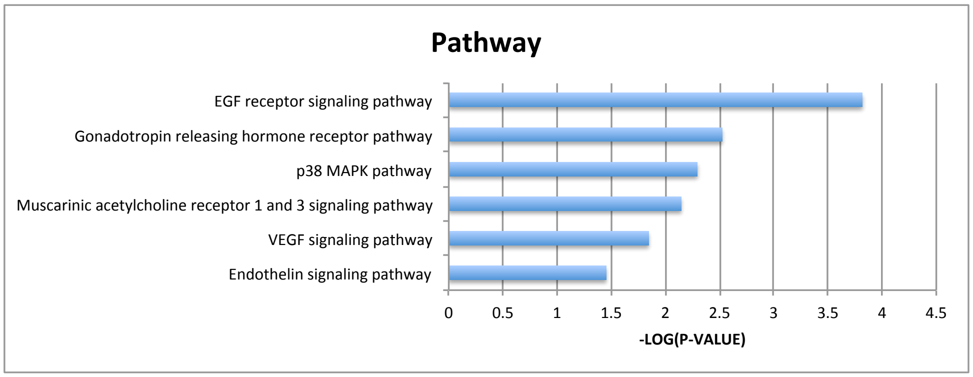

Figure 11: **Pathway enrichment in averaged modules that are differentially expressed either in CD or UC (unaffected tissues)**. Chart summarising the pathways that are enriched in the nodes associated with CD in the averaged subnetworks that are differentially expressed either in CD or UC. No pathways were found below this threshold in the nodes associated with UC. P-value threshold was set to 0.05.

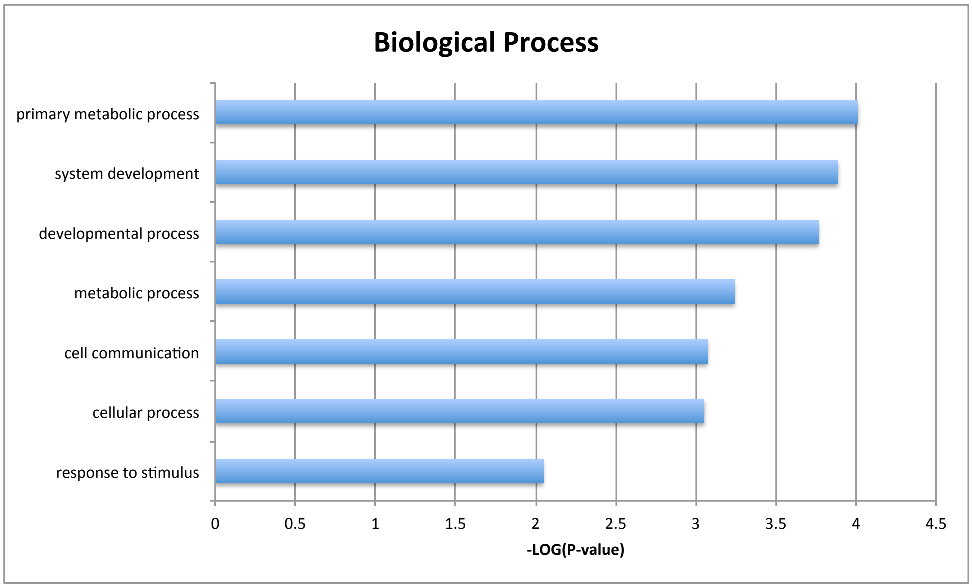

(a)

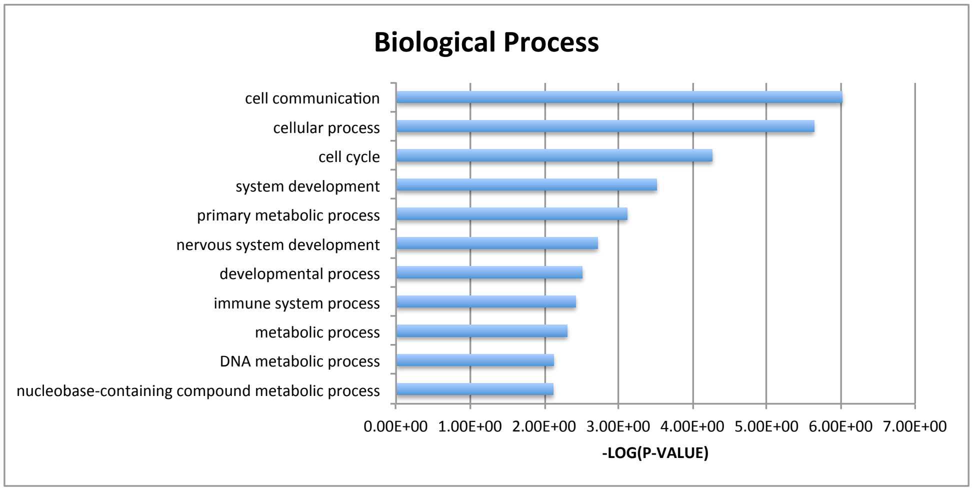

(b)

Figure 12: **Biological processes enrichment in averaged modules that are differentially expressed both in CD and UC**. Chart summarising the biological processes that are enriched in the nodes associated with CD and UC in affected (a) and unaffected (b) tissue in the averaged subnetworks that are differentially expressed both in CD and UC. P-value threshold was set to 0.01.

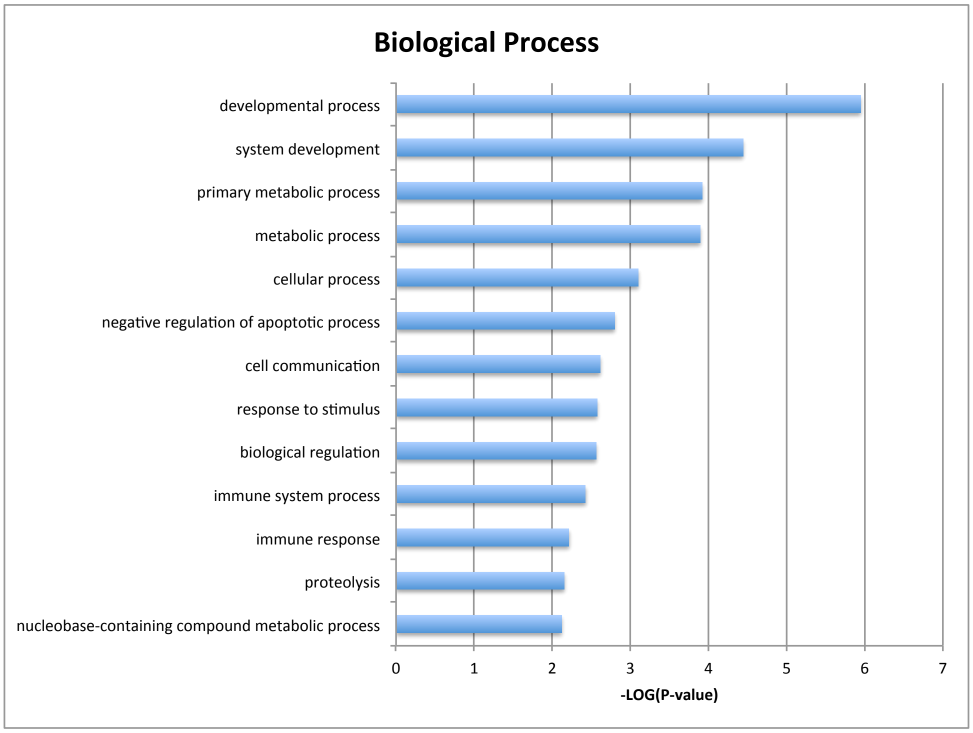

(a)

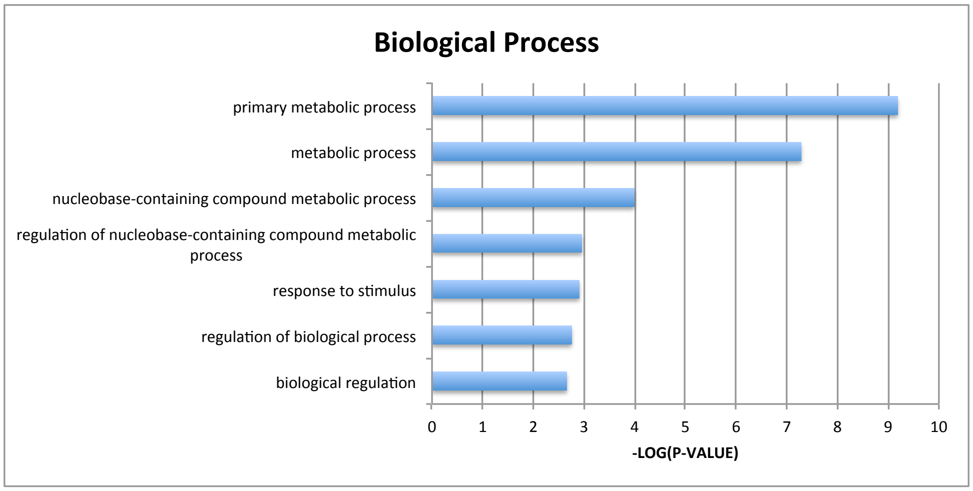

(b)

Figure 13: **Biological processes enrichment in averaged modules that are differentially expressed either in CD or UC (affected tissues)**. Chart summarising the biological processes that are enriched in the nodes associated with CD (a) and UC (b) in the averaged subnetworks that are differentially expressed either in CD or UC. P-value threshold was set to 0.01.

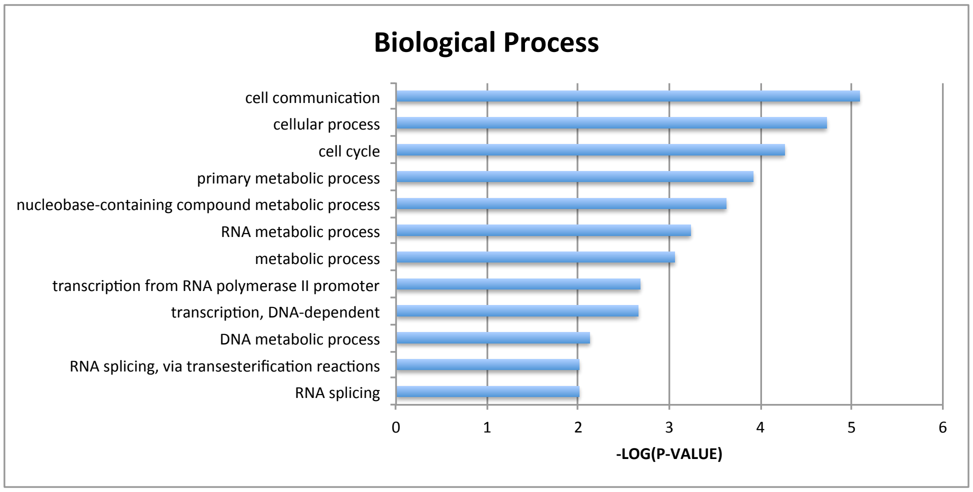

(a)

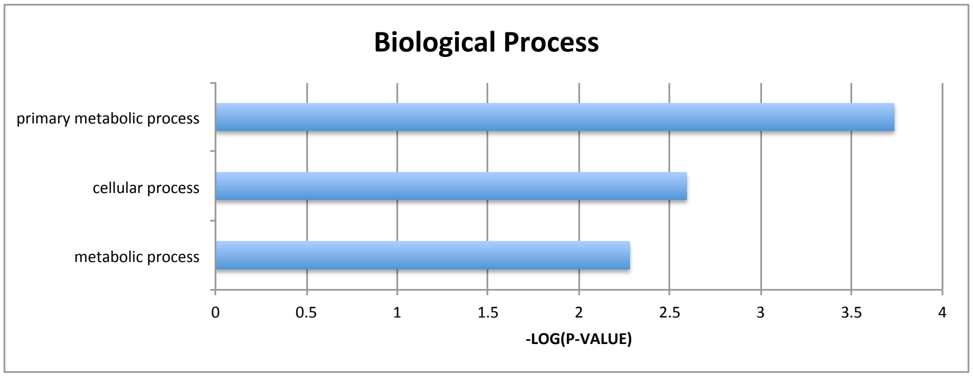

(b)

Figure 14: **Biological processes enrichment in averaged modules that are differentially expressed either in CD or UC (unaffected tissues)**. Chart summarising the biological processes that are enriched in the nodes associated with CD (a) and UC (b) in the averaged subnetworks that are differentially expressed either in CD or UC. P-value threshold was set to 0.01.

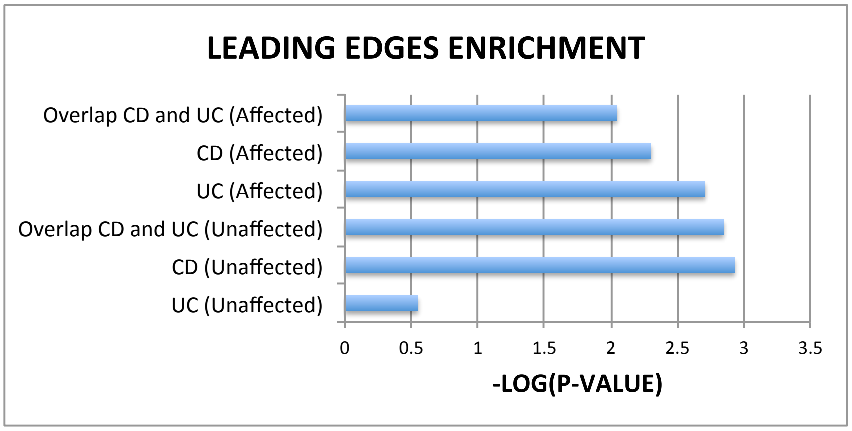

Figure 15: **Leading edges enrichment.** Enrichment of genes identified by our optimisation algorithm with leading edges found by GSEA. All p-values are minor than 0.01 except for when the algorithms are applied to microarray data derived from unaffected tissues in UC.

References

1. Lewis AC, Jones NS, Porter MA, Deane CM: **The function of communities in protein interaction networks at multiple scales**. *BMC Syst Biol* 2010 Jul 22; 4:100.

2. Pandey J, Koyutürk M, Subramaniam S, Grama A. **Functional coherence in domain interaction networks**. *Bioinformatics* 2008 Aug 15;24(16):i28-34.
